# Quality of life among colorectal cancer survivors participating in a pilot randomized controlled trial of a web-based dietary intervention with text messages

**DOI:** 10.1007/s00520-023-07620-x

**Published:** 2023-02-10

**Authors:** Lufan Wang, Crystal Langlais, Stacey A. Kenfield, Katherine Van Loon, Angela Laffan, Chloe E. Atreya, June M. Chan, Li Zhang, Isabel E. Allen, Christine Miaskowski, Yoshimi Fukuoka, Jeffrey A. Meyerhardt, Alan P. Venook, Erin L. Van Blarigan

**Affiliations:** 1 Department of Epidemiology & Biostatistics, University of California, San Francisco, San Francisco, CA USA; 2 Present Address: IQVIA, Durham, NC USA; 3 Department of Urology, University of California, San Francisco, UCSF, San Francisco, CA USA; 4 UCSF Helen Diller Family Comprehensive Cancer Center, University of California, San Francisco, San Francisco, CA USA; 5 Department of Medicine, University of California, San Francisco, San Francisco, CA USA; 6 Department of Physiologic Nursing, University of California, San Francisco, San Francisco, CA USA; 7 Dana-Farber Cancer Institute, Boston, MA USA

**Keywords:** Quality of life, Colorectal cancer, Cancer survivorship, Text messages, Dietary intervention

## Abstract

**Purpose:**

We aimed to estimate the effect of a 12-week web-based dietary intervention with text messages on quality of life (QoL) among colorectal cancer (CRC) survivors.

**Methods:**

Between 2017 and 2018, 50 CRC survivors were randomized (1:1) to receive a 12-week web-based dietary intervention with daily text messages or wait-list control. Health-related QoL was assessed using the European Organization for the Research and Treatment of Cancer (EORTC) Quality of Life Questionnaire–Core 30 (QLQ-C30) and colorectal quality of life module (QLQ-CR29) at baseline, 12, and 24 weeks. Within- and between-group mean changes in health-related QoL with 95% confidence intervals (CI) were calculated for both arms.

**Results:**

Compared to the controls, participants receiving the intervention had an improvement in emotional functioning (mean change: 14.3; 95% CI: 3.0, 25.6) at 12 weeks and social functioning (mean change: 13.8; 95% CI: 2.1, 25.5) at 24 weeks. A decrease of fatigue from baseline was also observed in the intervention arm (mean change: − 9.1; 95% CI: − 17.1, − 1.1) at 24 weeks. No other changes in QoL scores were associated with the intervention.

**Conclusion:**

CRC survivors randomized to receive a web-based dietary intervention with text messages experienced higher emotional and social functioning. Further study with a larger population may be warranted.

**Trial registration:**

clinicaltrials.gov, NCT02965521. Registered 16 November 2016, https://clinicaltrials.gov/ct2/keydates/NCT02965521

## Introduction

Colorectal cancer (CRC) is the third most commonly diagnosed cancer among men and women in the USA, with a 5-year survival rate of 65% [1]. As of 2018, it was estimated that over 1.3 million people are living with a CRC diagnosis in the USA [2].

Cancer survivors often deal with physical (e.g., fatigue, pain) and psychological (e.g., fear of recurrence, anxiety, depression) effects associated with cancer and its treatment [3–6]. In addition, CRC survivors may experience specific issues which are unique to this diagnosis, such as living with a stoma and bowel dysfunction [7]. Bowel dysfunction is common among colorectal cancer survivors [8]. For example, approximately 80–90% of rectal cancer patients who underwent sphincter-preserving surgery experience low anterior resection syndrome (LARS) with varied degree of severity [9]. The symptoms of LARS include fecal incontinence, urgency, and incomplete evacuation [9]. Nearly half of these patients continue to experience symptoms more than a decade after surgery [10, 11]. These post-cancer symptoms may have a significant long-lasting impact on the physical health, mental well-being, and quality of life (QoL) of CRC survivors [12–14].

Recent evidence suggests that a healthy diet after diagnosis of CRC may be associated with improvements in both survival and health-related QoL [15–19]. Our team previously reported longer survival after CRC diagnosis among those who reported health behaviors more consistent with the American Cancer Society (ACS) guidelines. The guidelines include maintaining a healthy body weight; regular physical activity; and a diet that includes vegetables, fruits, and whole grains [20]. Consumption of diets high in vegetables and fiber and low in red meat in particular may be associated with a lower level of fatigue and alleviate gastrointestinal symptoms related to CRC survivorship like diarrhea, bloating, and flatulence [21, 22]. Cross-sectional studies have reported that CRC survivors who met the recommendation of eating five or more portions of fruits and vegetables per day had significantly better health-related QoL compared to those who did not [23, 24]. In a randomized controlled trial among 223 CRC survivors, a 12-month dietary intervention that aimed to reduce the consumption of red/processed meat and refined grains was associated with significant improvements in QoL and depression compared to usual care control [25]. These studies show the promise of dietary interventions on improving health-related QoL among CRC survivors. However, research remains limited.

An increasing number of studies suggest that behavioral interventions using web and mobile technology are feasible and acceptable approaches to modify dietary behavior [26–28]. They can be largely automated and cost-effective, and therefore, these interventions may be more scalable compared to in-person studies or those with frequent telephone counseling [29, 30]. The effect of web-based dietary interventions on health-related QoL among CRC survivors is unknown.

The Survivor Choices for Eating and Drinking study (SUCCEED) was a pilot randomized controlled trial designed to determine the feasibility and acceptability of a 12-week web-based dietary intervention with text messages among CRC survivors [31]. The intervention was intended to help CRC survivors improve their diet quality, including increased intake of vegetables, whole grains, and fish; reduced intake of processed meat and sugar-sweetened beverages; and moderate alcohol consumption (if the patients chose to drink at all). QoL was a secondary endpoint in the SUCCEED trial. This paper aimed to estimate the effect of the intervention versus wait-list control on health-related QoL at 12 weeks (immediately post-intervention) and 24 weeks (12 weeks after the text message program ended).

## Materials and methods

Details of the SUCCEED trial protocol, findings of feasibility and acceptability of the intervention, and estimated change in diet have been published previously [31]. CRC survivors randomized to the intervention engaged more with text messages than the study website. Additionally, the intervention increased whole grain intake from baseline to 12 and 24 weeks [31]. As noted above, this paper aimed to estimate the effect of the intervention on health-related QoL at 12 and 24 weeks post-enrollment. Informed consent was obtained from all participants. The study was conducted in accordance with recognized ethical guidelines and approved by the Institutional Review Broad of the University of California, San Francisco (UCSF). This trial was registered at clinicaltrials.gov (NCT02965521).

### Study sample, recruitment, and randomization

Methods for the SUCCEED trial have been published previously [31]. Briefly, potential eligible individuals with a previous diagnosis of colon or rectal adenocarcinoma were identified through the gastrointestinal oncology clinic at UCSF between April 2017 and May 2018. The trial was also advertised on the web. Individuals were eligible to participate if they were not actively undergoing standard cytotoxic chemotherapy; were considered disease-free or had stable disease status at enrollment; were able to speak and read English; had access to mobile phone with the Internet and text messaging capabilities; and had regular access to the Internet, were able to navigate websites, and fill out forms on the web. Individuals who were already meeting four or more of the six target dietary behaviors (i.e., ≥ 5 servings of vegetables; ≥ 3 servings of whole grains per day; ≥ 2 servings of fish per week; limited intake of processed meat and alcohol; avoiding sweetened beverages) at screening were excluded. After screening and informed consent, 50 individuals were enrolled in the study. Eligible participants were randomized 1:1 to intervention or wait-list control using a block randomization method generated by a study biostatistician (LZ).

### Intervention

Following randomization, participants assigned to the intervention arm received print materials; a personalized report that included information on whether the participants currently met, almost met, or did not meet each of the six dietary targets based on the screening survey; access to the study website; and daily text messages for 12 weeks. Details of the study website and text messages, including examples, have been previously described [31]. Twenty-one of the 84 text messages (25%) asked the participants for a reply. Text messages stopped after 12 weeks, but intervention participants were able to continue to access the study website after the 12-week intervention.

### Wait-list control

Participants randomized to the wait-list control arm received print materials on diet after CRC at enrollment. They had the option to receive the intervention from 12 to 24 weeks after completing the 12-week assessment. Twenty-one of the 25 control participants opted to receive the intervention.

### Outcome measures

All participants were assessed at baseline, 12, and 24 weeks. Health-related QoL was accessed using the European Organization for the Research and Treatment of Cancer (EORTC) Quality of Life Questionnaire–Core 30 (QLQ-C30) [32] and colorectal quality of life module (QLQ-CR29) [33] administered online using the UCSF Research Electronic Data Capture (REDCap) system [34, 35].

The 30-item QLQ-C30 contains five functioning scales (physical, role, cognitive, emotional, and social functioning), three symptom scales (fatigue, pain, nausea, and vomiting), six symptom items (dyspnea, insomnia, appetite loss, constipation, diarrhea, financial difficulties), and a global health status/QoL scale. Scores for each scale/item range from 0 to 100. A higher score for the global health status/QoL represents a better QoL, and higher scores on the functional scales reflect better functioning. Higher scores on the symptom scales reflect a higher level of symptomatology/problems. The QLQ-C30 scoring manual [36] was used to calculate the scores. This paper focuses on change in global health status, the five functioning scales, as well as two symptom scales (fatigue; nausea; and vomiting) and three symptom items (appetite loss; constipation; diarrhea) that we hypothesized to be potentially related to diet.

The 29-item QLQ-CR29 is a disease specific instrument used to supplement the EORTC QLQ-C30 to assess health-related QoL in patients with CRC. This 29-item questionnaire contains four subscales (urinary frequency (UF), blood and mucus in stool (BMS), stool frequency (SF), body image (BI)) and 19 single items (urinary incontinence, dysuria, abdominal pain, buttock pain, bloating, dry mouth, hair loss, taste, anxiety, weight, flatulence, fecal incontinence, sore skin, embarrassment, stoma care problems, sexual interest (assessed using different questions for men and women), impotence, and dyspareunia). Patients were asked to indicate their symptoms for sexual interest items during the past 4 weeks and symptoms for all other items/scales during the past week. Responses were linearly transformed into a score ranging from 0 to 100 [37]. Higher scores reflect better functioning on the functional scales/single items. Higher scores on the symptom scales/single items reflect a higher level of symptomatology/problems. In this study, we focused on the four subscales (UF, BMS, SF, and BI) as well as five single items (bloating, taste, flatulence, fecal incontinence, and weight) that we hypothesized to be potentially related to diet.

### Statistical analysis

Demographic and baseline characteristics were summarized for all participants. Frequency distributions were used to summarize categorical measurements, while mean (standard deviation (SD)) and median (interquartile range (IQR)) were used to describe symmetric and skewed continuous measurements, respectively. In accordance with the CONSORT guidelines for pilot and feasibility trials [38], efficacy statistical tests were not conducted. Point estimates with 95% confidence intervals (CI) of mean differences from baseline to 12 weeks and 24 weeks for each arm and the difference in mean health-related QoL score change between the two arms from baseline to 12 and 24 weeks were calculated and reported. All statistical analyses were conducted using R (Version 4.0.2 (R Core Team (2020))).

## Results

The CONSORT flow was previously published [31]. In brief, 94 individuals were assessed for eligibility, and 50 individuals were randomized 1:1 to the intervention (*n* = 25) or wait-list control (*n* = 25). The most common reason of ineligibility was meeting four or more of the six target dietary behaviors (*n* = 14) at enrollment. Follow-up based on completion of the health-related QoL questionnaires was 88% at both 12 and 24 weeks in the intervention arm and 92% and 80% at 12 and 24 weeks in the control arm, respectively.

Table 1 shows the baseline characteristics of all participants (please see the trial’s main paper for baseline characteristics by arm).^31^ The median age was 55.4 years (IQR: 50.7, 62.3), and 34% of participants identified as men. Most of the participants (70%) identified as non-Hispanic white race; 12% identified as Hispanic ethnicity. Most participants (70%) had stage III cancer at diagnosis. With a range of 4 to 407 months, the median time since diagnosis among the participants was 24 months (IQR: 14, 41). Regarding target dietary behaviors, most of the participants (82%) met the target dietary behavior of limited alcohol intake, but only 8% of the participants met the target dietary behavior of more than 5 servings of vegetables per day at enrollment.

**Table 1:** Demographic characteristics, clinical factors, and baseline health behavior practices of 50 individuals with CRC participating in a pilot 12-week web-based dietary intervention with text messages

Characteristic	Participants (*n* = 50)
Age, years, median (IQR)	55 (51, 62)
BMI, kg/m^2^	26.3 (23.6–30.7)
Male, *n* (%)	17 (34%)
Race, *n* (%)	
American Indian or Alaska Native	1 (2%)
Asian	3 (6%)
Black/African American	2 (4%)
Hispanic, Latino, or Spanish origin	6 (12%)
Native Hawaiian or other Pacific Islander	2 (4%)
Non-Hispanic white	35 (70%)
Other	1 (2%)
College degree, *n* (%)	48 (96%)
Work full-time, *n* (%)	28 (56%)
Married, *n* (%)	34 (68%)
Cancer type, n (%)	
Colon cancer	33 (66%)
Rectal cancer	17 (34%)
Months since diagnosis, median (IQR)	24 (14, 41)
Tumor stage, *n* (%)	
I	6 (12%)
II	6 (12%)
III	35 (70%)
IV	3 (6%)
Ostomy at enrollment, *n* (%)	11 (22%)
Number meeting dietary recommendation at enrollment	
≥ 5 servings/d vegetables, *n* (%)	4 (8%)
No processed meat, *n* (%)	6 (12%)
≥ 3 servings/d whole grains, *n* (%)	8 (16%)
≥ 2 servings/week fish, *n* (%)	12 (24%)
No sugar-sweetened beverages, *n* (%)	15 (30%)
< 2 and < 1 alcoholic drink/d for men and women, respectively, *n* (%)	41 (82%)

Baseline characteristics by arms have been published previously [31]

### Health-related QoL

Results from the EORTC QLQ-C30 are shown in Table 2. The global health status/QoL score of our participants was relatively high with a median of 75 for both arms at enrollment. At 12 weeks, individuals in the intervention arm showed improvements in emotional functioning (mean change: 9.1; 95% CI: 2.2, 16.0) and cognitive functioning (mean change: 4.6; 95% CI 1.2, 7.9) from enrollment. The change in emotional functioning was different from change observed in the controls (between-group mean difference in emotional functioning: 14.3; 95% CI: 3.0, 25.6). However, these differences did not appear to be maintained at 24 weeks.

**Table 2:** Estimated effect of a web-based dietary intervention with daily text messages versus wait-list control on health-related QoL among CRC survivors (*N* = 50)

	Time point	Intervention			Control			Difference in mean change (95% CI)^a^
		*n*	Median (IQR)	Mean change (95% CI)	*n*	Median (IQR)	Mean change (95% CI)	
Global health status	Baseline	25	75.0 (58.3, 83.3)		25	75.0 (50.0, 83.3)		
	12 weeks	22	75.0 (66.7, 83.3)	− 0.4 (− 9.0, 8.2)	22	79.2 (50.0, 83.3)	− 2.3 (− 6.86, 2.31)	1.9 (− 7.6,11.4)
	24 weeks	22	75.0 (66.7, 83.3)	− 1.5 (− 9.9, 6.9)	20	66.7 (56.3, 85.4)	2.5 (− 2.9, 7.9)	− 4.0 (− 13.7, 5.7)
Functioning scale								
Physical functioning	Baseline	25	100 (93.3, 100)		25	100 (93.3, 100)		
	12 weeks	22	100 (93.3, 100)	0 (− 3.0, 3.0)	23	100 (93.3, 100)	− 2.3 (− 5.0, 0.4)	2.3 (− 1.6, 6.3)
	24 weeks	22	100 (92.1, 100)	− 1.0 (− 4.6, 2.6)	20	100 (93.3, 100)	− 1.0 (− 2.8, 0.8)	0 (− 3.9, 3.9)
Role functioning	Baseline	25	100 (66.7, 100)		25	100 (83.3, 100)		
	12 weeks	22	100 (100, 100)	2.7 (− 8.3, 12.8)	23	100 (83.3, 100)	2.2 (− 5.8, 10.1)	0.6 (− 12.7, 12.9)
	24 weeks	22	100 (93.3, 100)	1.5 (− 8.7, 11.7)	20	100 (79.2, 100)	− 0.8 (− 7.9, 9.4)	2.4 (− 10.6, 15.3)
Emotional functioning	Baseline	25	83.3 (75.0, 91.7)		25	83.3 (58.3, 83.3)		
	12 weeks	22	91.7 (75.0, 100)	9.1 (2.2, 16.0)	23	66.7 (41.7, 79.2)	− 5.2 (− 14.5, 4.1)	14.3 (3.0, 25.6)
	24 weeks	22	95.8 (83.3, 100)	9.9 (− 4.3, 15.4)	20	75.0 (62.5, 83.3)	2.6 (− 8.2, 13.5)	7.2 (− 4.7, 19.1)
Cognitive functioning	Baseline	25	83.3 (66.7, 100)		25	83.3 (66.7, 83.3)		
	12 weeks	22	91.7 (83.3, 100)	4.6 (1.2, 7.9)	23	83.3 (58.3, 100)	0 (− 6.5, 6.5)	4.6 (− 2.7, 11.7)
	24 weeks	22	91.7 (83.3, 100)	3.0 (− 1.3, 7.4)	20	66.7 (66.7, 87.5)	− 3.3 (− 12.0, 5.3)	6.4 (− 3.1, 15.8)
Social functioning	Baseline	25	83.3 (66.7, 100)		25	83.3 (66.7, 100)		
	12 weeks	22	100 (83.3, 100)	9.1 (− 2.0, 20.2)	23	100 (66.7, 100)	− 0.7 (− 6.7, 5.2)	9.8 (− 2.5, 22.2)
	24 weeks	22	100 (83.3, 100)	12.1 (2.1, 22.1)	20	91.7 (66.7, 100)	− 1.7 (− 8.3, 5.0)	13.8 (2.1, 25.5)
Symptom scales								
Fatigue	Baseline	25	11.1 (0, 33.3)		25	11.1 (11.1, 33.3)		
	12 weeks	22	16.7 (0, 30.6)	− 4.6 (− 10.4, 1.3)	23	22.2 (11.1, 33.3)	− 1.9 (− 8.0, 4.2)	− 2.6 (− 10.8, 5.6)
	24 weeks	22	11.1 (0, 22.2)	− 9.1 (− 17.1, − 1.1)	20	22.2 (8.3, 33.3)	− 5.0 (− 14.0, 4.0)	− 4.1 (− 15.8, 7.6)
Nausea and vomiting	Baseline	25	0 (0, 0)		25	0 (0, 0)		
	12 weeks	22	0 (0, 0)	− 0.8 (− 4.4, 2.8)	23	0 (0, 0)	0.7 (− 3.3, 4.8)	0 (− 5.2, 5.3)
	24 weeks	22	0 (0, 0)	− 0.8 (− 6.1, 4.6)	20	0 (0, 0)	0.8 (− 2.2, 3.9)	− 1.6 (− 7.6, 4.4)
Appetite loss	Baseline	25	0 (0, 0)		25	0 (0, 0)		
	12 weeks	22	0 (0, 0)	1.5 (− 5.7, 8.7)	23	0 (0, 0)	1.5 (− 5.4, 8.3)	0.1 (− 9.6, 9.7)
	24 weeks	22	0 (0, 0)	3.0 (− 10.1, 9.3)	20	0 (0, 0)	− 1.7 (− 7.8, 4.5)	4.7 (− 3.8, 13.2)
Constipation	Baseline	25	0 (0, 0)		25	0 (0, 33.3)		
	12 weeks	22	0 (0, 25.0)	4.5 (− 7.8, 16.9)	23	0 (0, 33.3)	− 2.9 (− 14.3, 8.5)	7.5 (− 8.9, 23.8)
	24 weeks	22	0 (0, 0)	1.5 (− 10.1, 13.1)	20	0 (0, 33.3)	− 6.7 (− 18.6, 5.3)	8.2 (− 8.0, 24.3)
Diarrhea	Baseline	24	0 (0, 33.3)		25	0 (0, 33.3)		
	12 weeks	22	0 (0, 33.3)	− 3.2 (− 15.8, 9.4)	23	0 (0, 33.3)	5.8 (− 1.3, 12.9)	− 9.0 (− 23.1, 5.2)
	24 weeks	22	0 (0, 33.3)	0 (− 6.8, 6.8)	20	0 (0, 33.3)	6.7 (− 2.9, 16.2)	− 6.7 (− 18.1, 4.8)

Health-related quality of life was measured using EORTC QLQ-C30. A higher score for the global health status represents a better QoL, and higher scores on the functional scales (physical, role, emotional, cognitive, and social functioning) reflect better functioning. Higher scores on the symptom scales (fatigue, nausea and vomiting, appetite loss, constipation, diarrhea) reflect a higher level of symptomatology/problems

^a^Difference in mean change was calculated by mean change from baseline in the intervention arm − mean change from baseline in the control arm

At 24 weeks, the intervention arm had a greater improvement in social functioning compared to the controls (between-group mean difference: 13.8; 95% CI: 2.1, 25.5); this difference was not observed at 12 weeks. Additionally, the intervention arm appeared to improve in fatigue at 24 weeks (mean different: − 9.1, 95%CI: − 17.1, − 1.1), but this change was not different from the mean change observed in the controls (between-group difference: − 2.6; 95% CI: − 10.8, 5.6). Participants had a median score of 0 for the nausea and vomiting, appetite loss, constipation, and diarrhea symptom subscales at enrollment.

The intervention was not associated with changes in disease-specific health-related QoL measured by EORTC QLQ-CR29 (Table 3). No change from enrollment was observed in any subscales or the 5 diet-related single items in the intervention arm at 12 or 24 weeks. However, participants had low symptom burden with all median scores clustered at the lower end of the scale at enrollment. Increases in scores, which indicate a higher level of problem, in blood or mucus in stool (mean change: 7.3; 95% CI: 2.5, 12.0), (concern about) body image (mean change: 12.1; 95% CI: 2.0, 22.2), and (concern about) weight (mean change: 14.5; 95% CI: 5.0, 24.0) were observed in the controls from 0 to 12 weeks, but there was no evidence of between-group differences in these symptoms.

**Table 3:** Estimated effect of a web-based dietary intervention with daily text messages versus wait-list control on aspects of disease-specific health-related QoL among CRC survivors (*N* = 50)

	Time point	Intervention			Control			Difference in mean change (95% CI)^a^
		*n*	Median (IQR)	Mean Change From baseline (95% CI)	*n*	Median (IQR)	Mean Change From baseline (95% CI)	
Urinary frequency	Baseline	25	16.7 (0, 33.3)		25	33.3 (16.7, 50.0)		
	12 weeks	21	16.7 (0, 33.3)	3.2 (− 5.4, 11.7)	23	16.7 (0, 41.7)	− 5.8 (− 12.8, 1.3)	9.0 (− 1.8,19.7)
	24 weeks	22	16.7 (0, 33.3)	2.3 (− 5.0, 9.6)	20	16.7 (0, 37.5)	− 5.8 (− 13.5, 1.9)	8.1 (− 2.2, 18.4)
Blood or mucus in stool	Baseline	25	0 (0, 0)		25	0 (0, 0)		
	12 weeks	21	0 (0, 0)	0.8 (-3.7, 5.3)	23	0 (0, 16.7)	7.3 (2.5, 12.0)	− 6.5 (− 12.8, 0.1)
	24 weeks	22	0 (0, 0)	7.6 (− 2.0, 3.5)	20	0 (0, 4.2)	1.7 (− 0.7, 4.1)	0.9 (− 4.5, 2.7)
Stool frequency	Baseline	25	16.7 (0, 33.3)		22	25.0 (0, 50.0)		
	12 weeks	19	16.7 (0, 33.3)	− 6.1 (− 14.3, 2.0)	23	33.3 (0, 50)	− 2.5 (− 10.2, 5.2)	− 3.6 (− 14.5, 7.2)
	24 weeks	21	0 (0, 16.7)	− 5.6 (− 16.6, 5.5)	19	16.7 (0, 41.7)	3.1 (− 7.2, 13.5)	− 8.7 (− 23.3, 5.9)
(Concern about) body image	Baseline	25	77.8 (55.6, 88.9)		25	66.7 (33.3, 100)		
	12 weeks	21	88.9 (77.8, 88.9)	9.5 (− 0.8, 19.8)	23	77.8 (55.6, 100)	12.1 (2.0, 22.2)	− 2.6 (− 16.6, 11.5)
	24 weeks	22	88.9 (69.4, 100)	6.1 (− 0.9, 13.0)	20	66.7 (33.3, 91.7)	3.3 (− 6.2, 12.9)	2.7 (− 8.7, 14.2)
Bloating	Baseline	24	0 (0, 33.3)		25	33.3 (0, 33.3)		
	12 weeks	21	0 (0, 0)	− 11.1 (− 25.0, 2.7)	23	33.3 (0, 33.3)	− 4.4 (− 14.4, 5.7)	− 6.8 (− 23.4, 9.9)
	24 weeks	22	0 (0, 33.3)	− 6.4 (− 16.7, 4.0)	20	0 (0, 33.3)	− 8.3 (− 19.5, 2.8)	2.0 (− 12.7, 16.7)
Taste	Baseline	25	0 (0, 0)		25	0 (0, 0)		
	12 weeks	21	0 (0, 0)	− 1.6 (− 7.4, 4.2)	23	0 (0, 0)	− 1.5 (− 8.3, 5.4)	− 0.1 (− 8.9, 8.6)
	24 weeks	22	0 (0, 0)	0 (− 9.1, 9.1)	20	0 (0, 0)	− 3.3 (− 14.5, 7.9)	3.3 (− 10.7,17.4)
Flatulence	Baseline	25	33.3 (0, 33.3)		22	33.3 (0, 33.3)		
	12 weeks	19	0 (0, 33.3)	− 7.0 (− 15.6, 1.6)	23	33.3 (0, 33.3)	6.7 (− 6.3, 19.7)	− 13.7 (− 28.8, 1.5)
	24 weeks	21	33.3 (0, 33.3)	0 (− 11.8, 11.8)	19	33.3 (33.3, 33.3)	10.4 (− 2.1, 22.9)	− 10.4 (− 27.0, 6.1)
Fecal incontinence	Baseline	25	0 (0, 0)		22	0 (0, 25.0)		
	12 weeks	19	0 (0, 0)	0 (− 7.6, 7.6)	23	0 (0, 33.3)	3.33 (− 1.5, 8.1)	− 3.33 (− 12.1, 5.4)
	24 weeks	21	0 (0, 0)	0 (− 6.8, 6.8)	19	0 (0, 16.7)	0 (0, 0)	0 (− 6.8, 6.8)
(Concern about) weight	Baseline	24	66.7 (33.3, 100)		25	66.7 (33.3, 66.7)		
	12 weeks	21	66.7 (33.3, 100)	5.0 (− 6.6, 16.6)	23	66.7 (50.0, 100)	14.5 (5.0, 24.0)	− 9.5 (− 24.1, 5.11)
	24 weeks	22	66.7 (33.3, 91.7)	0 (− 9.6, 9.6)	20	66.7 (25.0, 100)	5.0 (− 6.2, 16.6)	− 5.0 (− 19.6, 9.6)

Colorectal cancer specific health-related quality of life was measured using EORTC QLQ-CR29. Higher scores reflect better functioning on the functional scales/single items (weight, body image). Higher scores on the symptom scales/single items reflect a higher level of symptomatology/problems (urinary frequency, blood or mucus in stool, stool frequency, bloating, taste, flatulence, fecal incontinence)

^a^Difference in mean change was calculated by mean change from baseline in the intervention arm − mean change from baseline in the control arm

## Discussion

The purpose of this secondary analysis was to estimate the effect of a 12-week web-based dietary intervention with daily text messages on health-related QoL among CRC survivors. Participants receiving the intervention showed improvements in the emotional functioning subscale of EORTC QLQ-C30 at 12 weeks compared to participants in the control arm, though this did not result in an improvement in overall global health score.

Health-related QoL is an important outcome for successful cancer survivorship [39]. For CRC in particular, survivors often deal with health-related QoL challenges from physical, social, emotional, and cognitive factors [40, 41]. While data are limited, adherence to lifestyle recommendations, including a healthy diet, has been associated with higher QoL after CRC treatment [15, 25]. For example, a study from Kenkhuis et al. reported that higher dietary fiber, fruit, and vegetable intake was associated with better physical and role functioning and less fatigue in the first 2 years after CRC treatments [21]. However, adherence with these recommendations is suboptimal, especially dietary behaviors, and improving diet after diagnosis is one of the goals of CRC survivorship care [42].

A number of small studies have examined the effect of mobile health lifestyle interventions with websites, mobile apps, text messages, and activity trackers among cancer survivors [43–53]. Most of them have focused on physical activity or physical activity plus dietary interventions among breast cancer [47–50] or prostate cancer [51–53] survivors. No published studies have assessed the effect of technology-based diet-only interventions on HRQoL among CRC survivors. Thus, the present study adds to our understanding of the effects of web-based dietary interventions on health-related QoL in this population.

In our study, the magnitude of changes in emotional functioning, cognitive functioning, social functioning, and fatigue were clinically meaningful. According to the interpretation guide of EORTC QLQ-C30 [54], the changes we observed in emotional functioning at 12 weeks and social functioning at 24 weeks are categorized as medium improvements. The changes observed in cognitive functioning and fatigue were small improvements. Deficits in emotional and social functioning are factors hampering the QOL among CRC patients [13]. Thus, larger studies to confirm our observations may be warranted. In addition, there are no previous data available to estimate the SD of change scores for the EORTC QLQ-C30 among this population of colorectal cancer survivors. Thus, the sample size needed in future studies examining these outcomes could be calculated using the effect sizes from the interpretation guide [54] and the SD of the change scores from our study.

No intervention effects were observed for the other subscales, including physical and role functioning, nausea and vomiting, appetite loss, constipation, and diarrhea. It is worth noting that scores of these subscales in our study sample were relatively higher at baseline compared to general CRC survivors at similar age or stage [55]. This higher ceiling may limit the room for improvements. To better examine the effect of the intervention on physical aspects of QoL, a study population with more symptoms in these domains at enrollment is needed.

Strengths of this study include the randomized design and high retention rate (90% at 12 weeks). However, there are several limitations that should be noted. More than half (66%) of our participants were female, and most of the participants (96%) possessed college degrees and relatively high baseline health-related QoL, which may limit the ability to detect improvement as well as the generalizability of the study. Further, while 30% of our study sample identified as a race other than non-Hispanic white, we had low enrollment of Black CRC survivors—the group with the highest mortality rate from CRC [56]. It is also possible that the changes we observed in emotional and social functioning would not have been observed if we had used an attention control versus a wait-list control [57].

In conclusion, our results suggest a potential beneficial effect of a web-based dietary intervention on aspects of health-related QoL among CRC survivors and provide insights for future study planning. Future studies should focus on defining the optimal time to intervene and type of intervention, including duration and frequency of messages, enrolling a more diverse population with lower health-related QoL at enrollment, and determining the long-term sustainability of the intervention’s effects.

## Data Availability

De-identified data are available upon request.

## References

[1] Siegel RL, Miller KD, Fuchs HE, Jemal A (2022). Cancer statistics, 2022. CA Cancer J Clin.

[2] Cancer of the Colon and rectum - Cancer Stat Facts. SEER. Accessed February 11, 2022. https://seer.cancer.gov/statfacts/html/colorect.html

[3] Harrington CB, Hansen JA, Moskowitz M, Todd BL, Feuerstein M (2010). It’s not over when it’s over: long-term symptoms in cancer survivors—a systematic review. Int J Psychiatry Med.

[4] Gernier F, Joly F, Klein D, Mercier M, Velten M, Licaj I (2020). Cancer-related fatigue among long-term survivors of breast, cervical, and colorectal cancer: a French registry–based controlled study. Support Care Cancer.

[5] Götze H, Friedrich M, Taubenheim S, Dietz A, Lordick F, Mehnert A (2020). Depression and anxiety in long-term survivors 5 and 10 years after cancer diagnosis. Support Care Cancer.

[6] Inhestern L, Beierlein V, Bultmann JC (2017). Anxiety and depression in working-age cancer survivors: a register-based study. BMC Cancer.

[7] Han CJ, Yang GS, Syrjala K (2020). Symptom experiences in colorectal cancer survivors after cancer treatments: a systematic review and meta-analysis. Cancer Nurs.

[8] Denlinger CS, Barsevick AM (2009) The challenges of colorectal cancer survivorship. Robinson KG, ed. J Natl Compr Canc Netw 7(8):883–894 10.6004/jnccn.2009.0058 PMC3110673 19755048

[9] Nguyen TH, Chokshi RV (2020). Low anterior resection syndrome. Curr Gastroenterol Rep.

[10] Sturiale A, Martellucci J, Zurli L (2017). Long-term functional follow-up after anterior rectal resection for cancer. Int J Colorectal Dis.

[11] Chen TYT, Wiltink LM, Nout RA (2015). Bowel function 14 years after preoperative short-course radiotherapy and total mesorectal excision for rectal cancer: report of a multicenter randomized trial. Clin Colorectal Cancer.

[12] LeMasters T, Madhavan S, Sambamoorthi U, Kurian S (2013). A population-based study comparing HRQoL among breast, prostate, and colorectal cancer survivors to propensity score matched controls, by cancer type, and gender: HRQoL of breast, prostate, and colorectal cancer survivors. Psychooncology.

[13] Arndt V, Merx H, Stegmaier C, Ziegler H, Brenner H (2004). Quality of life in patients with colorectal cancer 1 year after diagnosis compared with the general population: a population-based study. JCO.

[14] Lloyd S, Baraghoshi D, Tao R (2019). Mental health disorders are more common in colorectal cancer survivors and associated with decreased overall survival. Am J Clin Oncol.

[15] Balhareth A, Aldossary MY, McNamara D (2019). Impact of physical activity and diet on colorectal cancer survivors’ quality of life: a systematic review. World J Surg Onc.

[16] Van Blarigan EL, Meyerhardt JA (2015). Role of physical activity and diet after colorectal cancer diagnosis. JCO.

[17] Meyerhardt JA, Niedzwiecki D, Hollis D (2007). Association of dietary patterns with cancer recurrence and survival in patients with stage III colon cancer. JAMA.

[18] Kassianos AP, Raats MM, Gage H, Peacock M (2015). Quality of life and dietary changes among cancer patients: a systematic review. Qual Life Res.

[19] Gigic B, Boeing H, Toth R (2018). Associations between dietary patterns and longitudinal quality of life changes in colorectal cancer patients: the ColoCare Study. Nutr Cancer.

[20] Van Blarigan EL, Fuchs CS, Niedzwiecki D (2018). Association of survival with adherence to the American Cancer Society nutrition and physical activity guidelines for cancer survivors after colon cancer diagnosis: the CALGB 89803/Alliance Trial. JAMA Oncol.

[21] Kenkhuis MF, van Duijnhoven FJB, van Roekel EH (2022). Longitudinal associations of fiber, vegetable, and fruit intake with quality of life and fatigue in colorectal cancer survivors up to 24 months post-treatment. Am J Clin Nutr.

[22] El-Shami K, Oeffinger KC, Erb NL (2015). American Cancer Society colorectal cancer survivorship care guidelines. CA Cancer J Clin.

[23] Grimmett C, Bridgewater J, Steptoe A, Wardle J (2011). Lifestyle and quality of life in colorectal cancer survivors. Qual Life Res.

[24] Blanchard CM, Courneya KS, Stein K (2008). Cancer survivors’ adherence to lifestyle behavior recommendations and associations with health-related quality of life: results from the American Cancer Society’s SCS-II. JCO.

[25] Ho M, Ho JWC, Fong DYT (2020). Effects of dietary and physical activity interventions on generic and cancer-specific health-related quality of life, anxiety, and depression in colorectal cancer survivors: a randomized controlled trial. J Cancer Surviv.

[26] Spring B, Duncan JM, Janke EA (2013). Integrating technology into standard weight loss treatment: a randomized controlled trial. JAMA Intern Med.

[27] Cadmus-Bertram L, Wang JB, Patterson RE, Newman VA, Parker BA, Pierce JP (2013). Web-based self-monitoring for weight loss among overweight/obese women at increased risk for breast cancer: the HELP pilot study. Psychooncology.

[28] Simpson SA, Matthews L, Pugmire J et al (2020) An app-, web- and social support-based weight loss intervention for adults with obesity: the HelpMeDoIt! feasibility RCT. NIHR Journals Library. Accessed February 8, 2022. http://www.ncbi.nlm.nih.gov/books/NBK555017/32186839

[29] Coughlin SS, Whitehead M, Sheats JQ, Mastromonico J, Hardy D, Smith SA (2015). Smartphone applications for promoting healthy diet and nutrition: a literature review. Jacobs J Food Nutr.

[30] Lumpkins CY, Mabachi N, Lee J, Pacheco C, Greiner KA, Geana M (2017). A prescription for internet access: appealing to middle-aged and older racial and ethnic minorities through social network sites to combat colorectal cancer. Health Commun.

[31] Van Blarigan EL, Kenfield SA, Chan JM (2020). Feasibility and acceptability of a web-based dietary intervention with text messages for colorectal cancer: a randomized pilot trial. Cancer Epidemiol Biomarkers Prev.

[32] Aaronson NK, Ahmedzai S, Bergman B (1993). The European Organization for Research and Treatment of Cancer QLQ-C30: A quality-of-life instrument for use in international clinical trials in oncology. JNCI J Natl Cancer Inst.

[33] Gujral S, Conroy T, Fleissner C (2007). Assessing quality of life in patients with colorectal cancer: an update of the EORTC quality of life questionnaire. Eur J Cancer.

[34] Harris PA, Taylor R, Thielke R, Payne J, Gonzalez N, Conde JG (2009). Research electronic data capture (REDCap)—a metadata-driven methodology and workflow process for providing translational research informatics support. J Biomed Inform.

[35] Harris PA, Taylor R, Minor BL (2019). The REDCap consortium: building an international community of software platform partners. J Biomed Inform.

[36] Fayers PM, Aaronson NK, Bjordal K, Groenvold M, Curran D, Bottomley A, on behalf of the EORTC Quality of Life Group (2001) The EORTC QLQ-C30 Scoring Manual (3rd Edition). Published by: European Organisation for Research and Treatment of Cancer, Brussels

[37] Whistance RN, Conroy T, Chie W (2009). Clinical and psychometric validation of the EORTC QLQ-CR29 questionnaire module to assess health-related quality of life in patients with colorectal cancer. Eur J Cancer.

[38] Eldridge SM, Chan CL, Campbell MJ et al (2016) CONSORT 2010 statement: extension to randomised pilot and feasibility trials. BMJ 355 10.1136/bmj.i5239 PMC5076380 27777223

[39] Morgan MA (2009). Cancer survivorship: history, quality-of-life issues, and the evolving multidisciplinary approach to implementation of cancer survivorship care plans. Oncol Nurs Forum.

[40] Shaw RD, Ivatury SJ (2021). Late and long-term symptom management in colorectal cancer survivorship. Dis Colon Rectum.

[41] Vardy J, Dhillon HM, Pond GR (2014). Cognitive function and fatigue after diagnosis of colorectal cancer. Ann Oncol.

[42] Winkels RM, van Lee L, Beijer S (2016). Adherence to the World Cancer Research Fund/American Institute for Cancer Research lifestyle recommendations in colorectal cancer survivors: results of the PROFILES registry. Cancer Med.

[43] Rees-Punia E, Leach CR, Westmaas JL, et al. Pilot randomized controlled trial of feasibility, acceptability, and preliminary efficacy of a web-based physical activity and sedentary time intervention for survivors of physical inactivity-related cancers. Int J Behav Med. Published online May 6, 2021 10.1007/s12529-021-09999-5 PMC8099708 33954891

[44] Koontz BF, Levine E, McSherry F (2021). Increasing physical activity in Cancer Survivors through a Text-messaging Exercise motivation Program (ICanSTEP). Support Care Cancer.

[45] Robertson MC, Lyons EJ, Liao Y, Baum ML, Basen-Engquist KM (2020). Gamified text messaging contingent on device-measured steps: randomized feasibility study of a physical activity intervention for cancer survivors. JMIR Mhealth Uhealth.

[46] Job JR, Eakin EG, Reeves MM, Fjeldsoe BS (2021). Evaluation of the healthy living after cancer text message-delivered, extended contact intervention using the RE-AIM framework. BMC Cancer.

[47] Lynch BM, Nguyen NH, Moore MM (2019). A randomized controlled trial of a wearable technology-based intervention for increasing moderate to vigorous physical activity and reducing sedentary behavior in breast cancer survivors: the ACTIVATE Trial. Cancer.

[48] Mcneil J, Brenner DR, Stone CR (2019). Activity tracker to prescribe various exercise intensities in breast cancer survivors. Med Sci Sports Exerc.

[49] Cairo J, Williams L, Bray L, Goetzke K, Perez AC (2020). Evaluation of a mobile health intervention to improve wellness outcomes for breast cancer survivors. J Patient Cent Res Rev.

[50] Phillips SM, Penedo FJ, Collins LM, et al. Optimization of a technology-supported physical activity promotion intervention for breast cancer survivors: results from Fit2Thrive. Cancer. Published online November 23, 2021 10.1002/cncr.34012 PMC8837679 34812521

[51] Chan JM, Van Blarigan EL, Langlais CS (2020). Feasibility and acceptability of a remotely delivered, web-based behavioral intervention for men with prostate cancer: four-arm randomized controlled pilot trial. J Med Internet Res.

[52] Sajid S, Dale W, Mustian K (2016). Novel physical activity interventions for older patients with prostate cancer on hormone therapy: a pilot randomized study. J Geriatr Oncol.

[53] Kenfield SA, Van Blarigan EL, Ameli N (2019). Feasibility, acceptability, and behavioral outcomes from a technology-enhanced behavioral change intervention (Prostate 8): a pilot randomized controlled trial in men with prostate cancer. Eur Urol.

[54] Cocks K, King MT, Velikova G (2012). Evidence-based guidelines for interpreting change scores for the European Organisation for the Research and Treatment of Cancer Quality of Life Questionnaire Core 30. Eur J Cancer.

[55] Fayers PM, Aaronson NK, Bjordal K, Groenvold M, Curran D, Bottomley A, on behalf of the EORTC Quality of Life Group (2001) The EORTC QLQ-C30 scoring manual, 3rd edn. European Organisation for Research and Treatment of Cancer, Brussels

[56] Siegel RL, Miller KD, Goding Sauer A (2020). Colorectal cancer statistics, 2020. CA Cancer J Clin.

[57] Aycock DM, Hayat MJ, Helvig A, Dunbar SB, Clark PC (2018). Essential considerations in developing attention control groups in behavioral research. Res Nurs Health.

